# Establishing a stable, repeatable platform for measuring changes in sperm DNA methylation

**DOI:** 10.1186/s13148-018-0551-7

**Published:** 2018-09-18

**Authors:** Mohammad Abbasi, Andrew D. Smith, Harish Swaminathan, Peer Sangngern, Amanda Douglas, Alan Horsager, Douglas T. Carrell, Philip J. Uren

**Affiliations:** 1 Episona, 69 N. Catalina Ave., Pasadena, USA; 2 University of Southern California, 1051 Childs Way, Los Angeles, 90089 USA; 3 University of Utah School of Medicine, 30 N 1900 E, Salt Lake City, 84132 USA; 4 National Genetics Institute, 2440 S Sepulveda Blvd, Los Angeles, 90064 USA

**Keywords:** DNA methylation, Epigenetics, Clinical Laboratory Improvement Amendments, CLIA, Laboratory-developed test, LDT, Male infertility

## Abstract

**Background:**

Several independent research groups have shown that alterations in human sperm methylation profiles correlate with decreased fecundity and an increased risk of poor embryo development. Moving these initial findings from the lab into a clinical setting where they can be used to measure male infertility though requires a platform that is stable and robust against batch effects that can occur between sample runs. Operating parameters must be established, performance characteristics determined, and guidelines set to ensure repeatability and accuracy. The standard for technical validation of a lab developed test (LDT) in the USA comes from the Clinical Laboratory Improvement Amendments (CLIA). However, CLIA was introduced in 1988, before the advent of genome-wide profiling and associated computational analysis. This, coupled with its intentionally general nature, makes its interpretation for epigenetic assays non-trivial.

**Results:**

Here, we present an interpretation of the CLIA technical validation requirements for profiling DNA methylation and calling aberrant methylation using the Illumina Infinium platform (e.g., the 450HM and MethylationEPIC). We describe an experimental design to meet these requirements, the experimental results obtained, and the operating parameters established.

**Conclusions:**

The CLIA guidelines, although not intended for high-throughput assays, can be interpreted in a way that is consistent with modern epigenetic assays. Based on such an interoperation, Illumina’s Infinium platform is quite amenable to usage in a clinical setting for diagnostic work.

**Electronic supplementary material:**

The online version of this article (10.1186/s13148-018-0551-7) contains supplementary material, which is available to authorized users.

## Background

Molecular diagnostics are shifting the way we evaluate and treat human disease. The rapid drop in costs to profile genomic information has resulted in the development of numerous molecular laboratory-developed tests (LDTs), particularly in cancer and reproductive health [1–5]. These molecular LDTs form the foundation of “personalized medicine,” an approach where detailed molecular information about an individual patient is collected to provide a more accurate diagnosis and, subsequently, targeted treatment [6–8].

In reproductive health, male factors contribute to the couple’s infertility in 50% of cases [9]. These male factors are primarily screened for through a semen analysis, which measures sperm concentration, motility, and morphology using light microscopy [10]. However, the semen analysis is a poor predictor of male fertility except in cases of extremely reduced sperm count [11, 12]. This is likely because sperm are responsible for fertilization and embryo development [13] issues that are molecular in nature. Thus, a molecular assay for measuring sperm quality would significantly improve our understanding of men’s reproductive health and allow for more targeted treatment of the infertile couple.

Over the last two decades, numerous studies have established that alterations in sperm DNA methylation (one of a number of epigenetic markers that promises potential clinical utility) are associated with poor reproductive outcomes [14–16]. Additionally, one study has shown that it may be possible to use the sperm epigenetic profile to predict the risk of poor fecundity and embryo development [12]. This has lead to the development of Episona’s fertility test. This test is a diagnostic assay for assessing (1) male fertility potential and (2) embryo development quality, two very important factors for successful pregnancy. It accomplishes this by measuring levels of DNA methylation at 485,000 locations (i.e., cytosine-phosphate-guanine (CpGs)) across the genome and comparing those methylation levels to those of the average fertile male (the average is taken from 156 semen samples from men with normal semen parameters and a history of at least 10 pregnancies). Methylation levels that are sufficiently different from the normal range at a given locus are reported as abnormal 1. We use Illumina’s 450K Human Methylation array (referred to throughout as the 450HM); however, the methodology used herein (but not the specific operating parameters obtained) is applicable to any similar technology.

To translate these findings into an assay that can be used clinically requires a technical validation of the platform. Although some initial steps have been taken to allow for the use of epigenetic information in the clinic [17–19], epigenetics-based LDTs must undergo technical validation (irrespective of their clinical utility) to show accuracy, precision, technical sensitivity, and technical specificity.

Generally, the regulatory framework for LDTs in the USA is guided by the Clinical Laboratory Improvement Amendments (CLIA). However, CLIA was not specifically designed to handle molecular tests that involve a significant computational component, and at the time of writing, the regulatory oversight of such LDTs is uncertain. The determination of whether a genomics-based LDT must conform to CLIA guidelines for technical validation or not is being performed on a case-by-case basis. Given the increase in prevalence for computationally complex genomics-based LDTs, additional regulatory guidance will likely be forthcoming at some point in the future. Irrespective of this though, at least for now, CLIA remains the most applicable guidance to frame a discussion of genomics-based LDT technical validation in the USA.

In this manuscript, we describe our interpretation of the CLIA requirements as they apply to epigenetic assays. In particular, we show a methodology for establishing precision, technical sensitivity, and technical specificity when employing Illumina’s Infinium-based assays for DNA methylation profiling that can be used beyond men’s reproductive health. We also determine reference intervals and the reportable range of methylation values in the specific case of sperm DNA methylation. To be clear, our interest is in the technical characteristics of the test and its underlying platform, not its clinical utility; clinical Laboratory Improvements Amendment (CLIA) does not consider clinical utility, and we will not explore it here either.

## Results

### Interpretation of CLIA for genome-wide sperm DNA methylation levels

The Clinical Laboratory Improvements Amendment (CLIA) is a regulatory instrument that applies to facilities within the USA that perform laboratory tests on human tissue intended for use in diagnosis, health assessment or disease treatment. The Center for Medicare and Medicaid Services (CMS) oversees CLIA and certifies laboratories that are in compliance. The legislation is dated to 1988 and so, not surprisingly, can be difficult to interpret in the context of more modern laboratory tests. Before delving into the experimental work we undertook for technical validation, we will explore the criteria set forth under CLIA and explain our interpretation in the context of DNA methylation and Illumina’s Infinium technology.

#### The analyte

The analyte is the substance whose presence is being identified or measured in the test. The original intention of CLIA and similar legislation was in regulating tests that measure an individual analyte in a sample. This can be either quantitatively or simply for the presence or absence of the analyte. A good example of a quantitive test is measuring a blood-alcohol level, while a test for the presence of a parasite would fall into the latter group.

Some personalized medicine tests can fit this mold relatively easily if they probe for the presence or absence of a relatively small number of features [8]. Panels of 10 or 20 single-nucleotide polymorphisms (SNPs) or gene-variants, for example, can be treated as ten or twenty independent tests for the presence of a specific nucleic acid.

The analyte we are measuring is DNA methylation. Illumina’s Infinium technology for profiling DNA methylation interrogates hundreds of thousands of individual CpG sites simultaneously [20]. Assuming the LDT may potentially utilize all of this information (or even a substantial fraction), it is not practical to treat these as independent measures that each require their own independent technical validation within the CLIA framework. We take the view that each measurement be treated as interchangeable measures of the same thing: DNA methylation level (the fraction of cells in the sample which are methylated at a given CpG, ranging from 0 to 1). We make an exception to this in the case of reference ranges, which we discuss more below.

Although we use the 450HM platform to measure methylation levels, the real purpose of the test is to call *aberrant methylation*. At a given locus, we are interested in whether the methylation level appears normal in a sample, or whether it falls outside what we would consider normal bounds. Sometimes, it makes more sense to think about the assay at this level. For example, when thinking about how repeatable the test is, it makes sense to look at how much the set of aberrant methylation calls changes between technical replicates—we are less interested in the (obviously related) variability of the underlying methylation levels. At other times, the methylation levels themselves will clearly be the more sensible level to consider. This happens for example when considering reference ranges, i.e., determining what is “normal.”

#### Criteria for assessment

CLIA defines six criteria that must be established by a laboratory for any LDT it performs (listed and briefly described in Table 1). The purpose of the criteria are not to lay down specific operating parameters for the test, but rather to ensure that the laboratory itself has undertaken the appropriate work to establish these parameters. Importantly, while manufacturer guidelines on instrumentation (or parameters established in other laboratories or studies) can be relevant for determining laboratory-specific performance specifications, they cannot be used as a replacement—analytical validation is specific to the lab in question. Hence, the requirements of CLIA are really more of a framework that the laboratory must work within to institute its own set of performance parameters. As a result, the CLIA criteria are broad and need to be interpreted in the context of the specific test being developed. Within this section, we put forward our interpretation for the specific case of measuring sperm DNA methylation levels using the Infinium technology and making aberrant methylation calls.

**Table 1:** CMS CLIA criteria for analytical validation of an LDT

Criteria	Description
Accuracy	Comparison of the test results to a gold-standard to determine how well it matches quantification or detection of the analyte.
Precision	Repeatability of the assay.
Analytical sensitivity	How well the test can detect low levels of the analyte.
Analytical specificity	How well the test detects the desired analyte and not other closely related contaminants
Reportable range	Upper and lower limit of levels that can reliably be reported; values outside the reportable range must be reported as either greater than the upper limit or less than the lower limit.
Reference interval	What is considered a “normal” outcome for the test.

The test we are describing measures DNA methylation levels, but it also makes a determination on the presence of an abnormal level, based on a normal reference range. Rather than ardently forcing one or both of these two measures on all of the criteria, we have selected the most natural fit for each of the criteria.

#### Determinations to be made and parameters to be established

Although CLIA specifies the criteria to be assessed, it gives only general guidance on what the substance of the assessment should be, or what should constitute the outcome. Based on this, and our own judgement, we propose three determinations to make: (1) to confirm that the performance characteristics of the platform are suitable for the intended application, (2) to establish acceptable performance ranges that can be monitored periodically, and (3) to determine operational parameters and inform standard operating procedures. Throughout the following sections evaluating the proposed criteria in Table 1, we will focus on addressing each of these three points.

We will now proceed to explain the experimental design to assess each criteria and present the results for technical validation of the Illumina Infinium platform under CLIA.

### Linearity and reportable range

When quantifying the presence of an analyte, the quantification must map linearly to the actual amount present in the sample. For particularly high or low levels, this may not be possible for certain analytes or measures. The range within which linearity is achieved is the reportable range. Values that fall outside this range must be reported as either higher than the maximum of the range or less than the minimum. The purpose of experiments here is to determine what this range is for the Infinium probes in the context of sperm DNA methylation levels.

Previous studies have shown that the Infinium technology, specifically the 450HM, is highly correlated with gold-standard methods such as pyrosequencing [21] and whole-genome bisulfite sequencing [20], with a strong linear relationship between gold-standard methylation levels and those reported by the assay. These studies did not establish specific thresholds for a reportable range however. Recall also that even if a method had been previously specified and thresholds determined, these experiments would need to be repeated and laboratory-specific ranges determined.

Our approach here was to collect whole-genome bisulfite sequencing (WGBS) and 450HM array methylation data from eight samples. Methylation values from these samples were then used to calculate the range where there is a linear relationship between methylation values from WGBS and 450HM array data using a previously described [22] linear regression procedure (details in the “Methods” section). In this approach, a series of samples with known values for the analyte are analyzed and the measured values are plotted on the *y* axis versus the known values on the *x* axis. The best straight line fit to the linear portion of the data is then used to find the reportable range of the test.

**Fig. 1:**
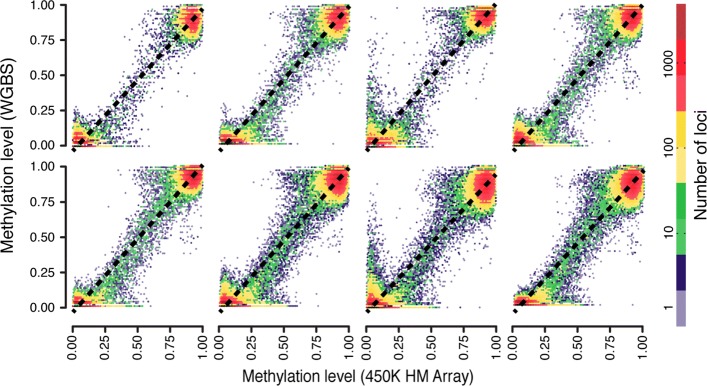
Linearity and reportable range. Heat map of methylation level for eight samples comparing methylation level reported from WGBS (*y*- xis) with methylation level reported from 450K Human Methylation array (*x* axis) at loci with at least 30X coverage in the WGBS

Heat maps of the methylation values from the WGBS and 450HM for each CpG site in the eight samples can be seen in Fig. 1. The linear models fitted to the data can also be seen on the figure. The low and high cut-offs for the linear range of the relationship between WGBS and 450HM array data is shown in Table 2. To get the overall reference range, we use the largest overlapping range for all the samples. This results in a linear reportable range of [0.052,1] for 450HM methylation values.

**Table 2:** Linearity and reportable range

Sample	1	2	3	4	5	6	7	8
High cut-off	1	1	1	1	1	1	1	1
Low cut-off	0.051	0.034	0.043	0.038	0.052	0.044	0.035	0.028
Adjusted R-squared	0.96	0.96	0.97	0.96	0.96	0.96	0.94	0.94

The high and low cut-off values for the linear range of each sample analyzed

**Fig. 2:**
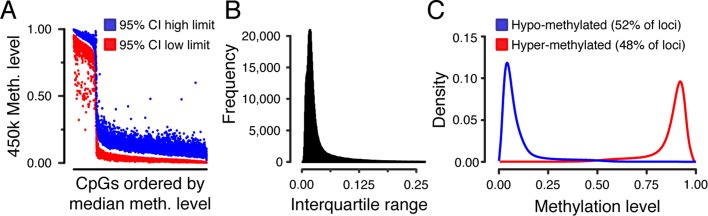
Reference range. **a** Reference interval for the methylation levels of 6690 CpG sites which have significant difference in methylation levels between fertile and infertile groups. Red and blue dots show the low and high limit for the 95% confidence interval respectively. **b** Distribution of the interquartile range for the methylation values of all CpGs in the 450HM array data for the known-fertile samples. **c** Density of methylation levels for hypo-methylated (blue) and hyper-methylated (red) loci in the fertile reference range exhibit unimodal distributions

While methylation levels below 0.052 cannot be exactly quantified, they can be bounded to be less than the linear range. This is sufficient for our needs, as we can still compute worst-case similarity to a reference level. It is worth noting that the low-methylation reference ranges we use are such that in almost all cases only values above the range can be considered aberrant (see Fig. 2a), so this has almost no practical impact for our application.

In terms of suitability for our purpose, we have no specific threshold that we require, but from a qualitative perspective, this is quite acceptable: close to 95% of the possible range is available to us. We can use this data to set operating parameters for the methylation levels we will report, but since we do not routinely run WGBS data, we cannot use this to set performance-monitoring parameters.

Although only eight samples were used in this analysis, population statistics collected from a larger group (156 samples; analysis presented in the following section) indicates that methylation levels across biological replicates have fairly low variance (see for example Fig. 2b, distribution of interquartile ranges). This in turn indicates that additional samples will behave in a similar fashion to these eight.

### Reference interval

The reference interval for a test refers to the expected “normal” range. This needs to be interpreted in the particular application. For our use, we interpret this as the empirical 95% confidence interval (i.e., an upper and lower limit which contains 95% of the values) on methylation levels at each CpG as measured from 156 methylomes profiled from known-fertile sperm donors (we also compute the maximum and minimum values, as well as the median, first, and third quartiles; however, it is the 95% confidence interval that we consider to define “normal”). The reference interval is one criteria where it is clearly necessary to consider each probe independently. Since the 450HM platform profiles approximately 480,000 CpG loci, we have approximately 480,000 such reference intervals. The upper and lower thresholds for a subset of these (at loci where sperm DNA methylation exhibits a correlation with fertility status—previously determined) are shown in Fig. 2a. Note that these loci are biased towards hypo-methylated regions.

Because we use the defined reference intervals to determine aberrant methylation, it is worth considering their properties more closely. Generally, the ranges are fairly tight, with the mode of the interquartile range distribution at approximately 0.03 (Fig. 2b), and 78% of the 95% confidence intervals less than 0.1. As a consequence, the distribution of methylation levels at individual CpGs is strongly unimodal, which can be seen by splitting all profiled CpGs into two groups, hypo- and hyper-methylated, by mean methylation level and plotting the distribution of methylation levels within each group (Fig. 2c).

We have established a conservative threshold for calling aberrant methylation on a per-CpG basis in an individual sperm methylome: either 0.2 above the upper 95% confidence interval threshold or 0.2 below the lower one. This allows methylation differences from the reference interval to be detected at 99.7% of loci profiled (in 0.3% of loci, the reference range is too large to allow differences to be detected). In 94.1% of cases where a methylation difference can be detected, the unimodal nature of the distributions ensures that a change in only one direction (hypo-methylated relative to reference interval or hyper-methylated relative to reference interval) is possible.

The 450HM technology was originally designed to aid in cancer studies [23], where both the tissue and the methylomes of the constituent cells are much more heterogeneous [24]. In our case, we are dealing with a purified cell type, with a much more sharply defined methylome. As a consequence, the criteria for aberrant methylation used here, while stringent, is nevertheless functional.

### Analytical sensitivity

The analytical sensitivity of a test refers to the limits at which the test can reliably detect or quantify the analyte. In the context of DNA methylation, we interpret this as the lower limit of DNA concentration for which reliable DNA methylation levels can be retrieved using the Infinium technology. Since the sample volume loaded is fixed at 15 *μ*l, concentrations are trivially convertible to amounts of DNA. Our goal is to set concentration thresholds for the samples to be run on the platform, below which samples will not be processed. We will also extrapolate from this minimum sperm cell-count thresholds to be applied on raw samples.

**Fig. 3:**
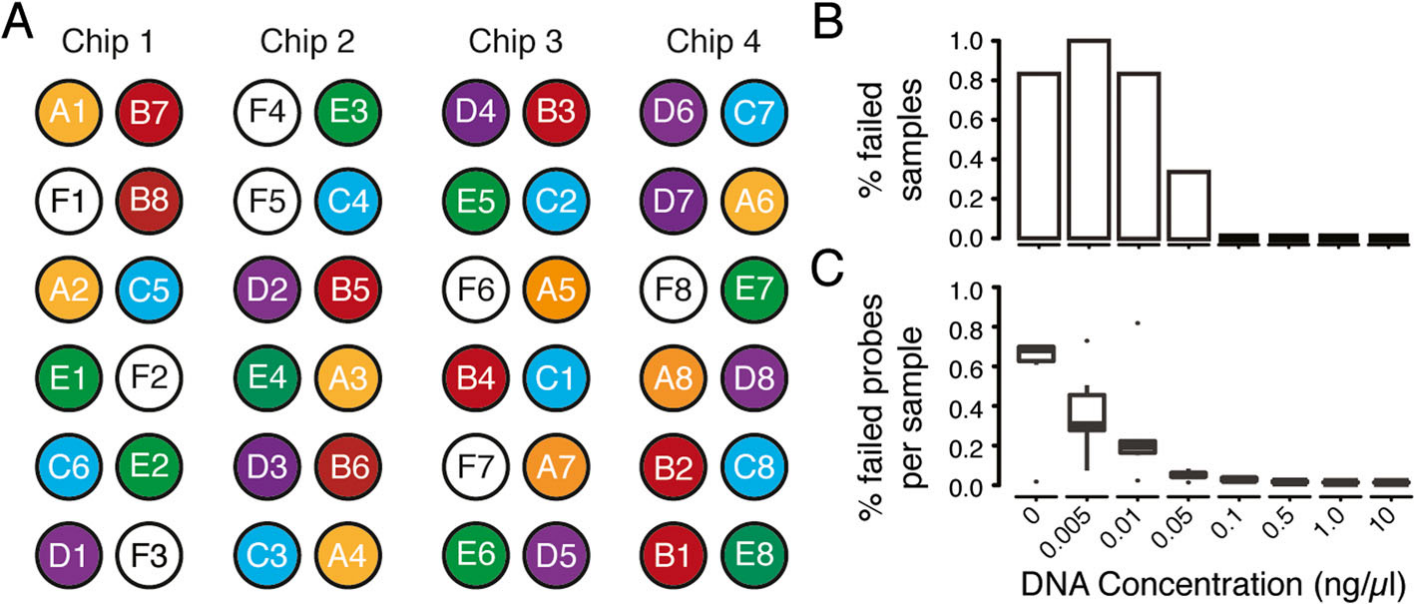
Analytical sensitivity experimental design and results. **a** Sample layout on 450HM chips. This experiment is looking at analytical sensitivity to various DNA concentrations. There are six different, color-coded samples (A, B, C, D, E, and F). Each sample contains eight different concentrations centered around the estimated limit of detection (LoD). Assuming the estimated LoD from previously collected data is 10 ng/ *μ*l, the concentrations will be blank (0 ng/ *μ*l), 0.005 ng/ *μ*l, 0.01 ng/ *μ*l, 0.05 ng/ *μ*l, 0.1 ng/ *μ*l, 0.5 ng/ *μ*l, 1 ng/ *μ*l, and 10 ng/ *μ*l (standard concentration). **b** The percentage of failed samples at varying DNA concentrations. **c** The percentage of failed probes per sample at varying DNA concentrations

Our approach is to process samples with varying concentration of DNA using the 450HM array and evaluate the success. The starting DNA sample is diluted into 8 values close to the limit of detection (LOD) for 6 biological samples (a total of 48 measurements). The LOD estimate was determined based on previously analyzed samples. To avoid potential technical confounds due to sample placement on the 450HM chip, or chip-level batch effects, we stratified these samples over four chips in different physical arrays. Figure 3a shows the stratification of the samples on the 450HM chips.

We then determine the number of probes that were unable to detect DNA levels above background (detection *p* value greater than 0.01; details in Methods section), and the number of samples considered failed (more than 5% failed probes).

The number of failed samples and the percentage of failed samples at each DNA concentration can be seen in Table 3 and Fig. 3b. The boxplot of the percentage of failed probes per sample for each concentration can be seen in Fig. 3c. Based on these results, we conservatively establish the DNA-concentration threshold for the fertility test at 0.5 ng/ *μ*l. This corresponds to 7.5 ng total DNA, post bisulfite conversion. Assuming a 90% loss from bisulfite conversion, this corresponds to approximately 75 ng of input DNA. With an expected 3pg of DNA per haploid sperm cell, this is around 25,000 cells. Again, conservatively, we establish our lower limit of sperm cell-count for samples at 1 million.

**Table 3:** Analytical sensitivity

Concentration (ng/ul)	0	0.005	0.01	0.05	0.1	0.5	1	10
Number of failed samples	5	6	5	2	0	0	0	0
Number of passed samples	1	0	1	4	6	6	6	6

The number of failed samples at each DNA concentration

### Precision

Simply put, precision refers to repeatability. Here, we are not concerned about whether the results of the test are accurate or not, but simply whether they are repeatable. Recall that CLIA does not set specific thresholds for precision nor specify how it should be measured, it is up to the laboratory to determine what is appropriate for the intended application, confirm that the technologies involved meet these requirements, and then continue to monitor precision. Since the test is concerned primarily with reporting on the presence of aberrant methylation at a set of CpGs, the most logical measure of repeatability is how similar the normal/abnormal calls are when running a sample multiple times. Before considering what is acceptable, let us first define our measure of similarity. Consider a single CpG. Given *n*_*j*_ technical replicates for sample *j*, the methylation level at this single CpG may be considered aberrant in all, some, or none of the replicates. Intuitively, “all” or “none” are good outcomes, while a 50/50 split of aberrant and normal calls is the worst. The number of replicates we call aberrantly methylated at a given CpG will depend on how stringent we are with our threshold, *t*. Recall that a methylation level is considered aberrant if it is more than *t* units distant from the normal 95% confidence interval. Let us then define the similarity of calls at CpG *i* for technical replicates of sample *j*, given threshold *t* as

1 $$ S_{ijt}=max (x_{ijt},n_{j}-x_{ijt})/n_{j},  $$

where *x*_*ijt*_ is simply the count of technical replicates for sample *j* called aberrantly methylated at CpG *i* given threshold *t*. The value *S*_*ijt*_ will range between 1 (perfect agreement) and 0.5 (the threshold perfectly bisects the methylation calls). We can average across all CpGs to get an overall value for a given sample at a given threshold as follows:

2 $$ S_{jt}=\frac{\sum_{i}S_{ijt}}{m},  $$

where *m* is the number of CpGs profiled. This is the empirical probability that another technical replicate for this sample will give an aberrant/normal methylation call at any given CpG that is consistent with the previous calls.

**Fig. 4:**
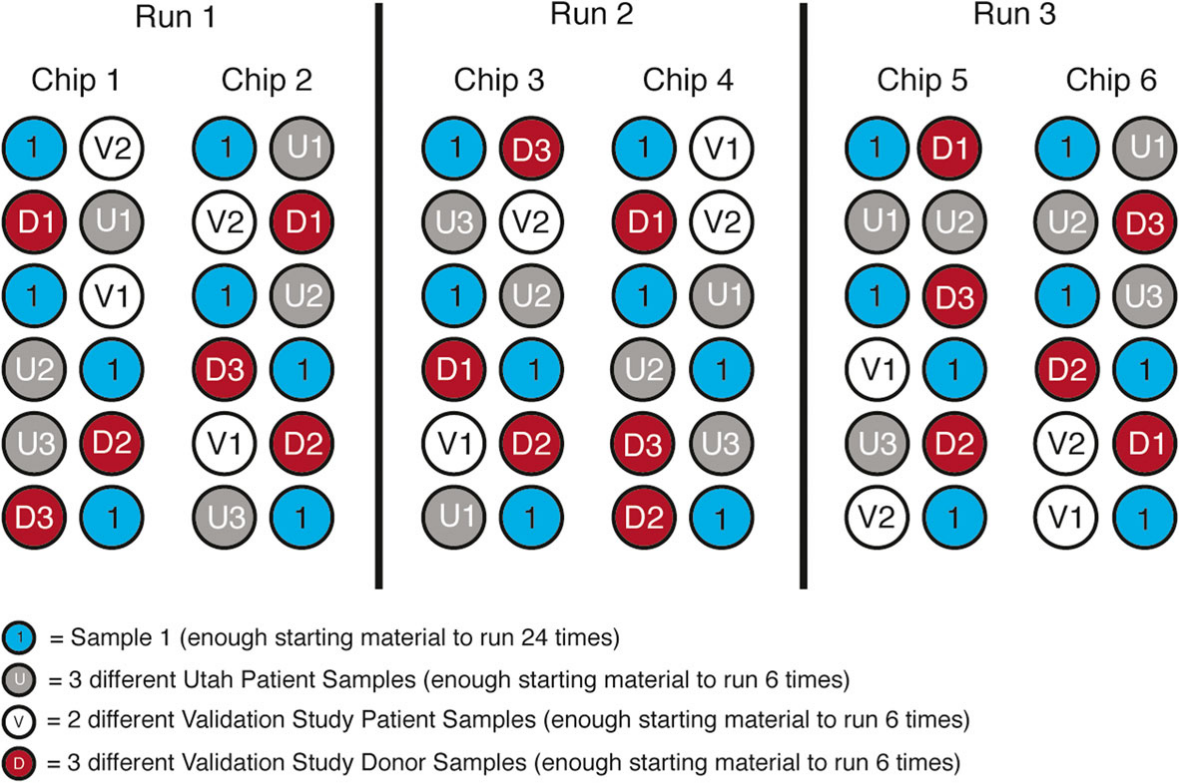
Precision experimental design. The stratification of the samples analyzed for precision on different chips and varying positions on chips

**Fig. 5:**
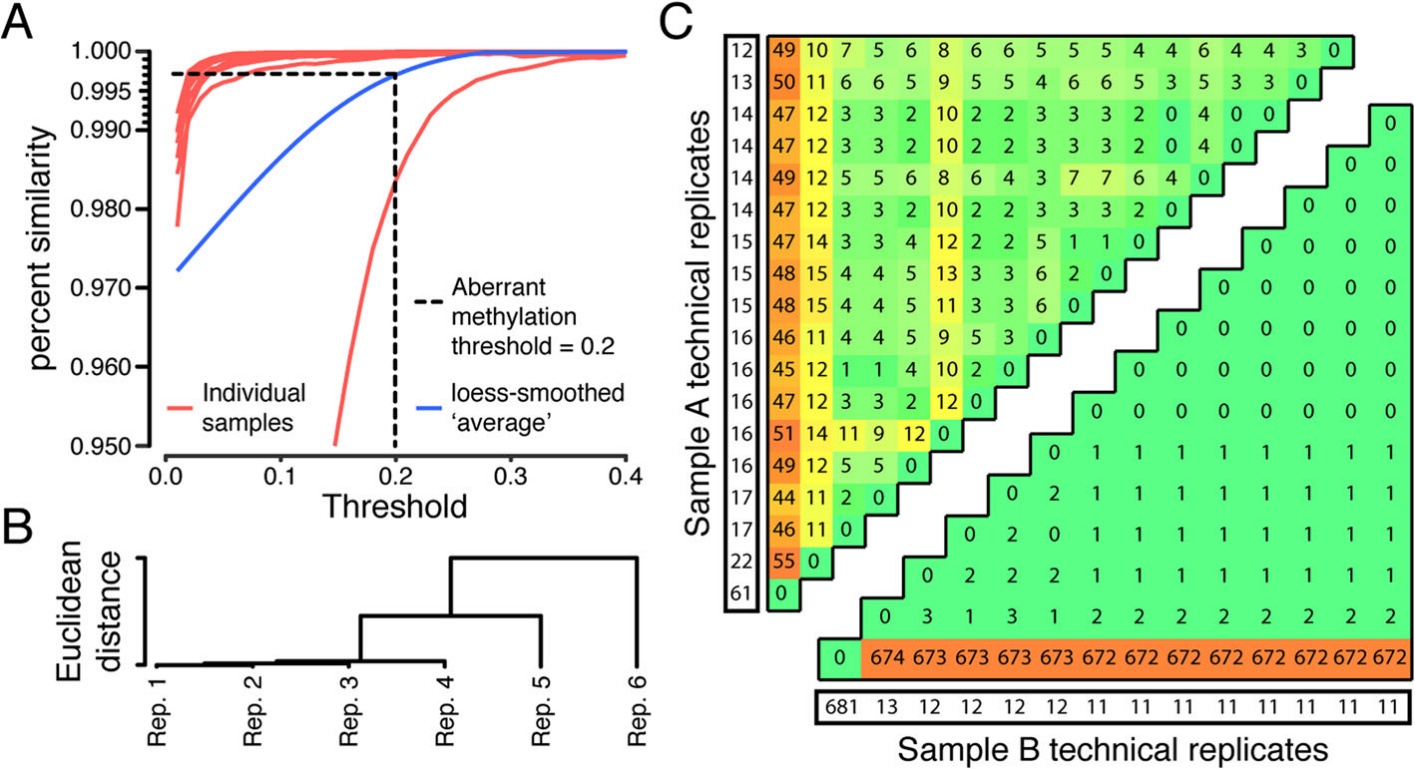
Precision results. **a** Empirical probability that a new replicate will give an aberrant methylation call consistent with existing replicates at different thresholds (of difference from fertile 95% confidence interval) for aberrant methylation calls. Dashed line shows the threshold used, which corresponds to an average probability of 0.997. **b** Euclidean distance between technical replicates of outlier sample from panel A. **c** Heatmap of the number of loci with different aberrant methylation status between technical replicates for two samples. Upper triangular matrix shows one sample and lower triangular matrix shows the other. Each row/column represents one technical replicate, and the cell at the intersection of a row/column is the number of differences between the sets of aberrantly methylated loci in the two replicates. The diagonal is the replicate compared with itself. Marginal cells (colored white) give the number of aberrantly methylated loci called in the corresponding replicate

We have six samples for profiling precision and have arranged technical replicates of these samples across three separate runs with six total chips. Samples are stratified across runs, chips, and positions to limit the impact of batch effects; the experimental design is shown schematically in Fig. 4, while Fig. 5a shows how *S*_*jt*_ varies as a function of the threshold *t* in each of the samples, as well as averaged across all samples. One outlier sample is clearly visible. We investigated this sample for obvious technical failure, but found none. The lower similarity is caused by two technical replicates that are outliers to the other four—see Fig. 5b, which shows the Euclidean distance between technical replicates for this sample. We have included this sample in our calculations as we feel it legitimately reflects possible technical variability. At a threshold of 0.2, the mean *S*_*jt*_ from all samples shows a value above 0.997 (highlighted in Fig. 5a). Put another way, we can expect a new normal/abnormal methylation call to be inconsistent with previous replicates on average less than 0.3% of the time, or about 1 in 333 calls.

We have two additional samples that we have run over a longer period of time both for further validation of our above observations and to assess whether inter-run variability over long periods is more noticeable. The first of these samples was run 14 times on separate days evenly spaced over 26 weeks, while the second was run 18 times over 34 weeks following the same schedule. We called the presence or absence of aberrant methylation in these replicates as previously described at 6690 candidate loci. Given any two replicates from the same sample, we can compute the dissimilarity in aberrant methylation calls as the number of loci called in one or the other replicate, but not both (i.e., the cardinality of the set difference). We show these values for all pairwise combinations in Fig. 5c. At a rate of 1 difference per 333 calls as calculated above, we expect less than 20 differences on average, which is borne out by the figure. Also noticeable is the presence of one outlier replicate for each sample that does not satisfy the expected rate, again relatively consistent with our observations on the previous six samples assayed.

The main utility of the precision analysis is in setting acceptable performance guidelines for proficiency testing (PT), which is a regular re-testing of previous samples to ensure consistency of results. We will consider an individual replicate to have passed PT if the number of differences in aberrant methylation calls does not exceed the 0.3% rate established above by more than 20%. An acceptable proficiency testing outcome will be 80% or better samples meeting this requirement.

### Analytical specificity

This criteria is concerned with whether the assay is specific to the substance that it intends to measure, or whether the presence of other substances causes erroneous results. Given that we ship samples at ambient temperature for several days, the most likely contaminant in our case is probably bacterial DNA. Hence, we will profile the impact of *E. coli* DNA contamination.

**Fig. 6:**
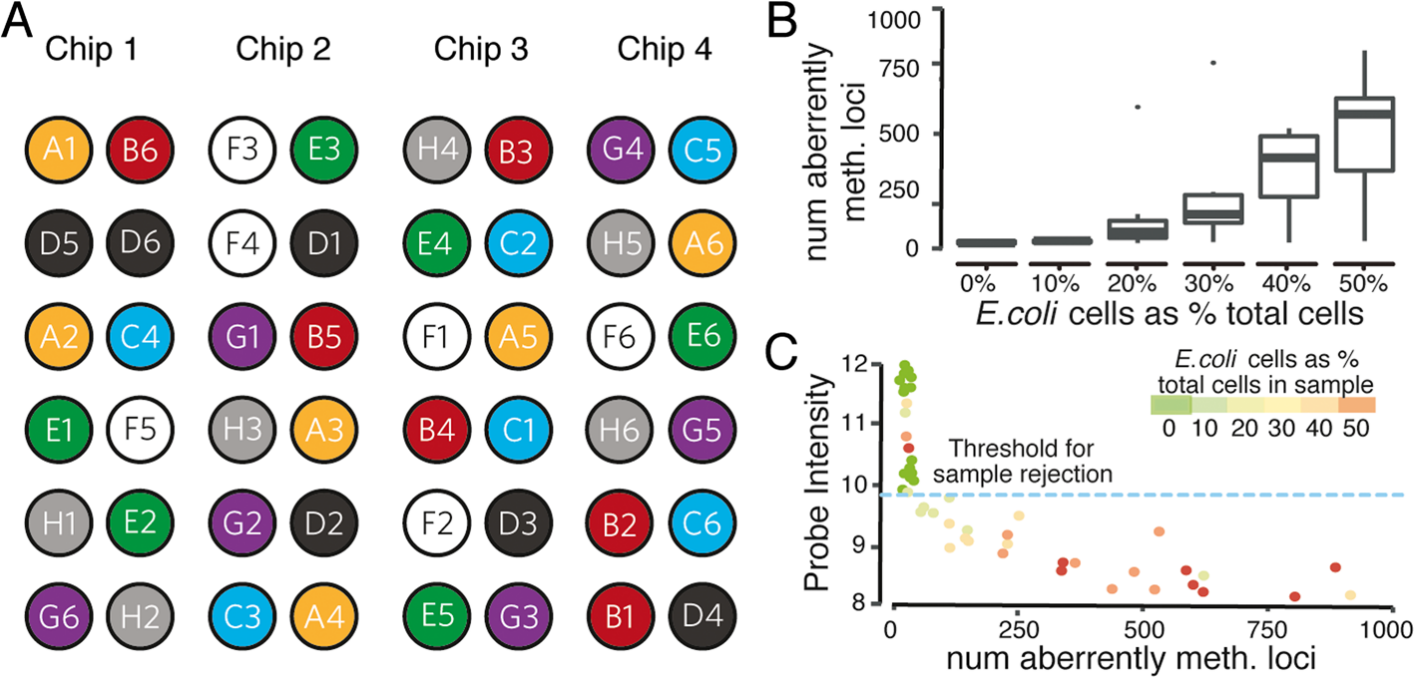
Analytical specificity. **a** The stratification of samples with different contamination levels on the 450HM array. There are eight different, color-coded samples labeled A through H. Each initial sample is divided into six replicates, which are contaminated with six different levels of bacterial cells; for example, A1 = no bacterial contamination, A2 = 10% of cells in sample are bacterial in origin, A3 = 20% of cells, A4 = 30% of cells, A5 = 40% of cells, and A6 = 50% of cells. **b** The number of aberrantly methylated loci per sample for different concentrations of bacterial cells (*E. coli* DNA) in the sample. **c** Scatter plot of the measure of probe intensity vs. the number of aberrantly methylated loci (failed probes are removed before calling aberrant methylation) per sample, stratified by the amount of bacterial contamination in the sample

We prepared 8 samples with enough starting material to be divided into 6 replicates each (for a total of 48 samples). For each sample, we add contaminating *E. coli* DNA (after the somatic cell lysis and wash step but before the bisulfite conversion step), at varying percentages to its six replicates: 0%, 10%, 20%, 30%, 40%, and 50% of the total cell count for the sample. These samples are arranged on four 450HM chips as per Fig. 6a, stratified to ameliorate any potential confound from sample placement.

To check the impact of contamination, we follow procedures used in the analytical sensitivity section to detect failed samples. The boxplot for the percentage of failed probes per sample for different contamination levels is shown in Additional file 1: Figure S1. We can see that the percentage of failed probes per sample remains under the 5% threshold for sample rejection for all the contamination levels of 40% or less. Two samples with contamination level of 50% have more than 5% of failed probes per sample and are labeled as failed.

These results suggest that either all the non-failed samples correctly detect the sample DNA, or their failure is not detected by looking at background control probes. Figure 6b shows that the number of aberrantly methylated loci generally increases as the level of bacterial contamination in the sample increases. This suggests that there are erroneous methylation level calls that are not detected by comparing to the control probes for background luminescence. This is due to the fact that there is an overall decrease in probe intensities, including background control probes, related to the increase in bacterial contamination (see Additional file 2: Figure S2).

To detect this kind of failure, we look at the median probe intensity of a sample to see whether it differs from what is expected from a normal run. We define probe intensity as [25]:

3 $$ MU_{j}=log_{2} \sqrt{M_{j} \times U_{j}},  $$

where *M*_*j*_ and *U*_*j*_ are the median methylated and unmethylated probe intensity values of sample *j*. We then need to define a threshold for probe intensity to detect failed samples.

Figure 6c shows high correlation between probe intensity and the number of aberrantly methylated loci for the replicates. This is used to choose a threshold for probe intensity by defining a normal range for the number of aberrantly methylated loci. We use replicates with no bacterial contamination to do this and define the 99% confidence interval of the number of aberrant calls for these replicates as the normal range and label any sample outside this range as failed. The threshold is chosen as the probe intensity that linearly separates and maximizes the margin between the two groups of failed and non-failed samples. From our experiment, this threshold is calculated as *MU*_*j*_=9.96 (blue line in Fig. 6c). Samples with probe intensity lower than the threshold are labeled as failed. Note that this separates the two groups with perfect accuracy in our data.

The scatter plot in Fig. 6c shows the relation between probe intensity and the number of aberrantly methylated loci called per replicate, where we split replicates into groups based on the amount of bacterial contamination introduced. We can see that replicates with high levels of bacterial contamination often had hundreds of aberrant calls and probe intensities lower than the threshold for failed samples. These replicates are detected and filtered out as failed samples. We also observed that the number of aberrant calls for non-failed samples with probe intensities higher than the threshold was low and close to numbers we expect from control samples with no bacterial contamination. Moreover, we observed no significant correlation between the number of aberrant methylation calls for non-failed samples and the amount of bacterial contamination (*p* value = 0.52).

### Accuracy

Of all the criteria, accuracy is probably the most intuitive. Here, the goal is to determine either how well the test reproduces known values from reference samples or how closely it compares to a gold-standard method. In this case, it makes most sense to consider the methylation level estimates from the 450HM directly.

We will use whole-genome bisulfite sequencing [26] as our reference gold-standard method for methylation level. We have eight samples where both WGBS and 450HM assays were run (the same samples that were used for profiling linearity and reportable range). CpG loci for comparison were selected to meet the following criteria: (1) coverage by the 450HM platform and (2) WGBS coverage greater than 30 but less than 100 reads (see the “Methods” section for justification). Correlation between WGBS and 450HM on a per-sample basis is visualized using heat-maps and quantified using Pearson’s product-moment correlation between the methylation values for WGBS and 450HM array data. Figure 1 shows the heat-maps comparing methylation levels measured via WGBS and 450HM in all eight samples. The correlation for each of the eight samples can be seen in Table 4. The mean correlation of the eight samples was 0.98 (sd = 0.01).

**Table 4:** Accuracy

Sample number	1	2	3	4	5	6	7	8
Correlation	0.98	0.98	0.98	0.98	0.98	0.98	0.97	0.97

Pearson’s correlation for each of the eight samples tested for 450HM accuracy

Since we will not routinely be running WGBS data, the comparison does not inform our standard operating procedures, nor parameters for performance monitoring. The main utility is determining whether the 450HM produces estimates of methylation level that are sufficiently accurate for our purpose. Again, we have no specific threshold for necessary accuracy, so our evaluation will be a qualitative one: methylation levels reported from the 450HM show very high similarity to WGBS levels, which we assess to be sufficient.

## Discussion

**Fig. 7:**
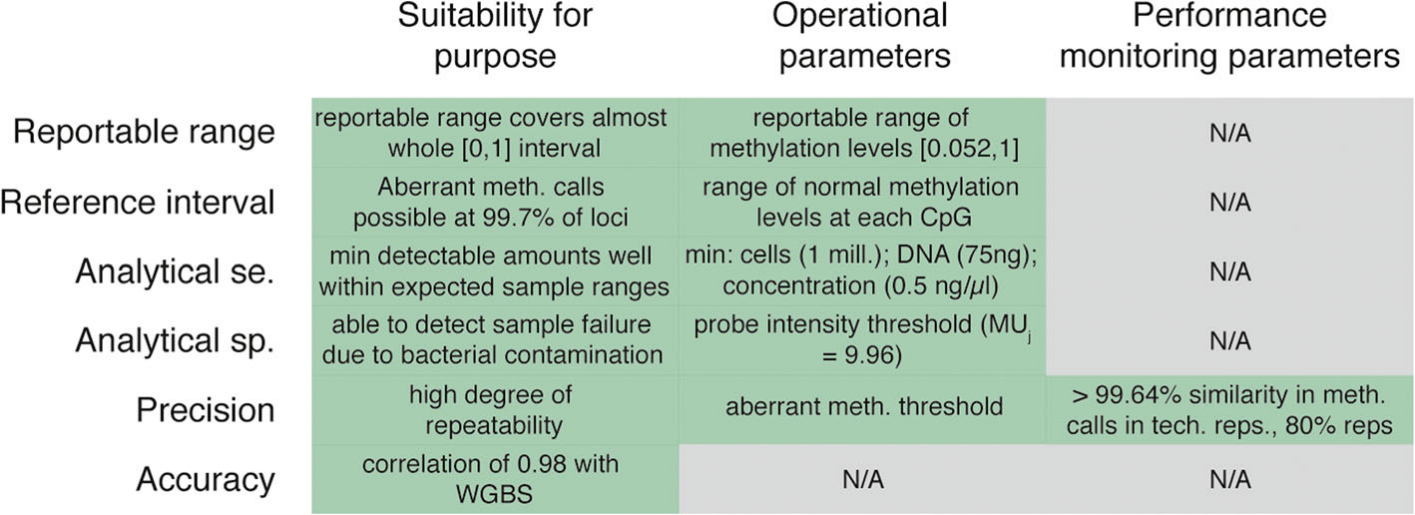
Summary of criteria. This matrix summarizes the findings of this study in each of the experiment groups in terms of the platform’s suitability for the intended use, the selection of operational parameters and the identification of criteria for continued performance monitoring

Our goal in this paper has been to provide a technical validation for an epigenetic LDT aimed at identifying aberrant methylation in sperm based on Illumina’s Infinium technology (specifically the 450HM in this instance) for profiling human methylation levels. To this end, we have used the CLIA regulatory requirements as a guiding framework, adopting its six criteria for assessment: reportable range, reference interval, analytical sensitivity, precision, analytical specificity, and accuracy. Within each of these, we have aimed to evaluate the platform’s suitability for the test, establish operating parameters and determine key indicators of performance for continued monitoring. Figure 7 gives a summary of the criteria assessed and the determinations made in each case.

We have established that the 450HM produces high-fidelity measurements of methylation levels in human sperm samples by comparing it to the widely accepted gold-standard method of whole-genome bisulfite sequencing. The correlation between the two methods is very high, with an average Pearson correlation of 0.98 (sd = 0.01), and consistently high correlation across multiple replicates with distinct biological properties. The 450HM platform produces reliably linear quantification of methylation levels between 0.052 and 1; this is the reportable range to be used in the fertility test.

Using multiple DNA concentrations, we have established that the 450HM assay reliably quantifies methylation levels at DNA concentrations of 0.5 ng/ *μ*l or above; this defines the threshold of DNA concentration to process a sample for the fertility test and by extension minimum total DNA and sperm cell counts.

We have demonstrated that the assay is highly reproducible. When using a 0.2 difference from reference range, the empirical probability that an additional replicate will produce a consistent aberrant methylation call at any given CpG is 0.997. This rate was confirmed with a long-running experiment testing technical replicates from two samples every two weeks over a period of more than 6 months.

By examining the impact of bacterial contamination, which is likely the most common contaminant to be faced when processing samples for the fertility test, we have demonstrated that our quality control procedures are capable of detecting samples with excessive bacterial contamination as failed.

Finally, we have given a summary of the reference interval compiled for the expected normal methylation levels at each of the CpG loci profiled by the 450HM platform.

## Conclusions

Although discussion is ongoing in terms of appropriate oversight and regulation of high-throughput clinical genomic and epigenetic diagnostic testing, CLIA remains probably the best regulatory guidance available. Although not specifically designed to deal with such testing, CLIA’s general nature allows (with some effort) an interpretation that is sensible in light of newer technologies. While regulatory bodies continue to grapple with appropriate oversight, it is important for the community to establish its own standards to both inform this process and maintain quality of service. Additionally, and more specifically, our evaluation of Illumina’s infinitum-based technology for epigenetic profiling shows that it is relatively stable and suitable for clinical epigenetic work, as long as appropriate quality control procedures are established and followed. The assay requires approximately 1 week for sample processing at-scale, including data analysis, and so is well within the range of many clinical tests.

## Methods

### Whole-genome bisulfite sequencing data-processing

WGBS data are used as a gold-standard for methylation values [26]. WGBS is based on the treatment of the DNA with sodium bisulfite which changes the unmethylated cytosine molecules into uracil molecules. Whole-genome sequencing is then used to determine the sequence of the treated DNA [27]. The WGBS data was processed using the following steps. The FastQ format reads were trimmed using the trimgalore [28] package and mapped to hg19 using Walt [29]. Bisulfite conversion rate was estimated using chrM and BGI’s control virus. Here, the bsrate function form the methpipe package was used to estimate the bisulfite conversion rate [30]. Afterwards, the duplicate-remover [30] function was used to remove duplicates and methylation levels were obtained on a per-site basis using methcount [30]. In the final step, information from symmetric CpGs were merged and CpG sites were filtered using the symmetric-cpgs [30] function.

### 450HM data-processing

The 450HM array data are collected using Ilumina’s 450HM beadchip array technology [20] and translated into methylation beta values using the minfi package in R [31]. The minfi package also allows us to perform subset-quantile within array normalization (SWAN) [32] to normalize the methylation beta values between the type 1 and type 2 probes. The resulting beta values are used as the 450HM array estimates of methylation level.

Determination of failed probes and samples is also performed using minfi. The package compares the total combined methylated and unmethylated DNA signal at each CpG site to the background-signal levels of the control probes on each array. The intensity of background probes is assumed to follow a normal distribution. A detection *p* value is computed for each locus based on this background distribution. Probes with a detection *p* value greater than 0.01 are considered to be failed probes. Samples with more than 5% failed probes are considered to be failed samples. These thresholds are consistent with previous studies [33].

### Subsetting of loci for 450HM versus WGBS comparison

The human genome has approximately 27 million CpG sites that can be methylated. WGBS data theoretically informs the methylation level at all of these. Due to the way methylation levels are inferred from WGBS data though (the fraction of reads covering a locus reporting methylation), loci with low coverage (few next-generation sequencing reads) have low resolution [20]. For example, with three reads covering a given CpG, the possible methylation levels reported are 0, 0.33, 0.66, and 1.0. To avoid poor correlations from this discretization, we do not use those loci with less than 30 reads covering them in this comparison [34]. Similarly, methylation levels derived from loci with very many more WGBS reads than expected have an increased risk of suffering from technical artifacts associated with the assay. Hence, we also do not use loci with more than 100 reads covering them in this comparison. These thresholds on average remove 83% of the common CpG sites between WGBS and 450HM array methylation data for a given sample. After filtering using the above thresholds, each pairwise comparison between WGBS and 450HM is based on an average of 82,011 loci.

### Linear model fitting for reportable range

To calculate the reportable range for each sample, we fit a linear model to the data [22]. The model has the following form:

4 $$ y_{i}=a+bx_{i}+e,  $$

where *y*_*i*_ is the WGBS methylation value and *x*_*i*_ is the 450HM methylation value for CpG site *i*. The intercept of the model is *a*, and the coefficient (which represents the slope of the fitted line) is *b*. Using the coefficient and intercept of the linear model, the low and high cut-off values for the linear reportable range for each sample are calculated by finding *x*_*i*_ when *y*_*i*_=0 and *y*_*i*_=1, respectively, bounded by 0 and 1. More formally:

5 $$\begin{array}{*{20}l} R_{low}&=(x_{i}|y_{i}=0)=\max(0,-a/b) \end{array} $$

6 $$\begin{array}{*{20}l} R_{high}&=(x_{i}|y_{i}=1)=\min(1,1-a/b) \end{array} $$

## Additional files

Additional file 1 **Figure S1.** The percentage of failed probes per sample for different concentrations of bacterial cells (*E. coli* DNA) in the sample. (TIF 938 kb)

Additional file 2 **Figure S2.** The probe intensity per sample for different concentrations of bacterial cells (*E. coli* DNA) in the sample. (A) Control probes in the red channel, (B) control probes in the green channel, (C) all probes in the red channel, and (D) all probes in the green channel. (TIF 3587 kb)

## Abbreviations

450HM: 450k human methylation
BGI: Beijing Genome Institute
CLIA: Clinical laboratory improvements amendment
CMS: Center for medicare and medicaid services
CpG: Cytosine-phosphate-guanine
DNA: Deoxyribonucleic acid
LDT: Laboratory developed test
LOD: Limit of detection
PT: Proficiency testing
SNP: Single-nucleotide polymorphism
SWAN: Subset-quantile within array normalization
WGBS: Whole-genome bisulfite sequencing

## Footnotes

1 These abnormalities are filtered for clinical relevancy, but since this filtering is not impacted by the technical stability of the underlying laboratory procedure for measuring DNA methylation levels, we will omit details of it here.
