# Supplementary figures and images for: Establishing a stable, repeatable platform for measuring changes in sperm DNA methylation
Source: Clin Epigenetics. 2018 Sep 18;10:119. doi: 10.1186/s13148-018-0551-7 (PMC6145208; doi:10.1186/s13148-018-0551-7)

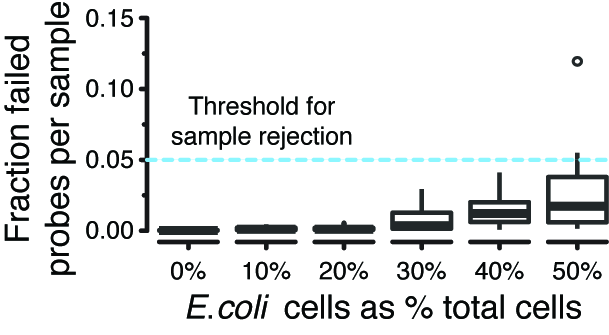

Supplement: Supplementary file 1 — Figure S1. The percentage of failed probes per sample for different concentrations of bacterial cells (E. coli DNA) in the sample. (TIF 938 kb) [file 13148_2018_551_MOESM1_ESM.tif]

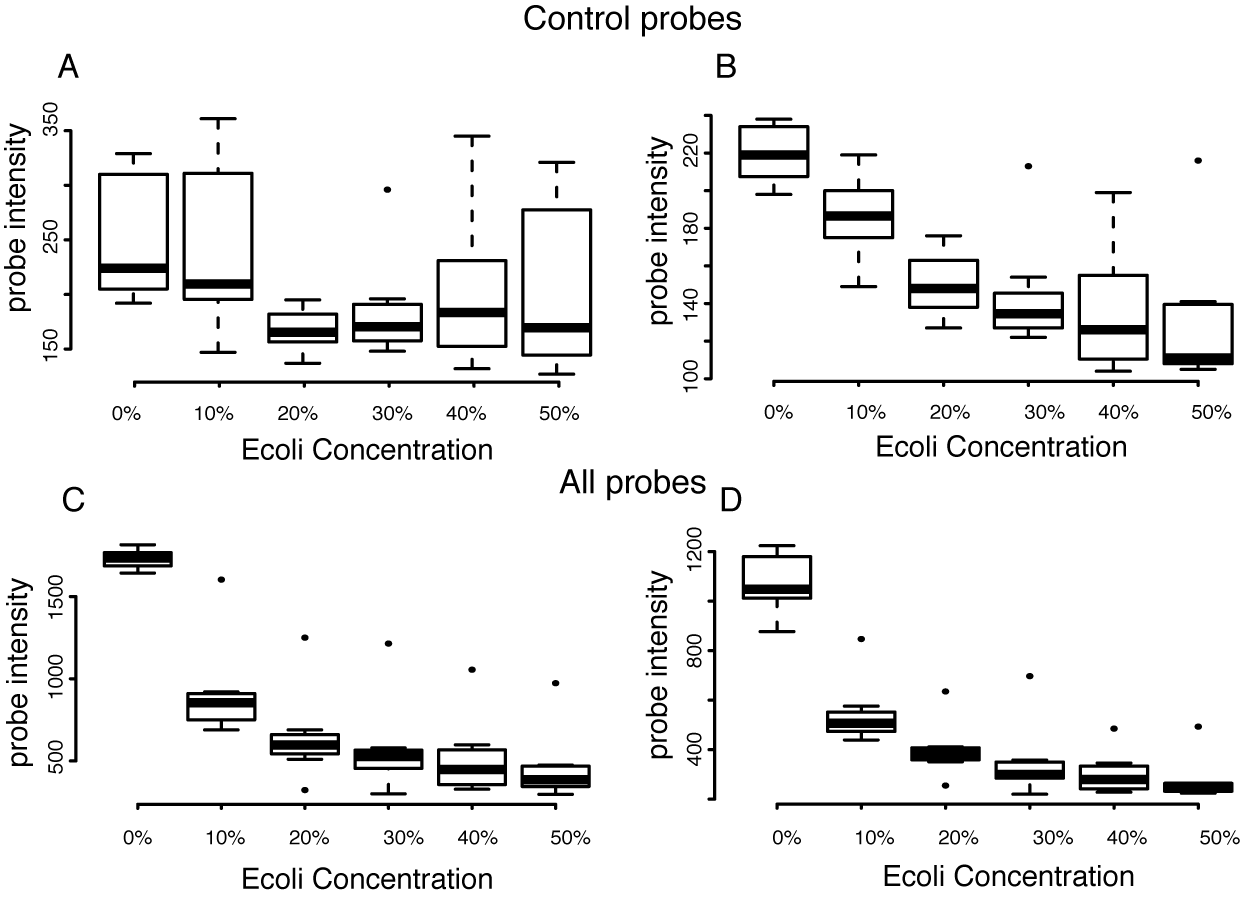

Supplement: Supplementary file 2 — Figure S2. The probe intensity per sample for different concentrations of bacterial cells (E. coli DNA) in the sample. (A) Control probes in the red channel, (B) control probes in the green channel, (C) all probes in the red channel, and (D) all probes in the green channel. (TIF 3587 kb) [file 13148_2018_551_MOESM2_ESM.tif]
